# Variational Autoencoders for Generative Drug-Gene Interactions in Periodontal Bone Resorption

**DOI:** 10.7759/cureus.65886

**Published:** 2024-07-31

**Authors:** Pradeep Kumar Yadalam, Ramya Ramadoss, Raghavendra Vamsi Anegundi

**Affiliations:** 1 Periodontics, Saveetha Dental College, Saveetha Institue of Medical and Technical Sciences (SIMATS) Saveetha University, Chennai, IND; 2 Oral Pathology and Oral Biology, Saveetha Dental College, Saveetha Institue of Medical and Technical Sciences (SIMATS) Saveetha University, Chennai, IND

**Keywords:** periodontitis, alveolar bone resorption, genes, drugs, variational autoencoders

## Abstract

Introduction

Periodontal bone resorption is a significant dental problem causing tooth loss and impaired oral function. It is influenced by factors such as bacterial plaque, genetic predisposition, smoking, systemic diseases, medications, hormonal changes, and poor oral hygiene. This condition disrupts bone remodeling, favoring resorptive processes. Variational autoencoders (VAEs) can learn the distribution of drug-gene interactions from existing data, identify potential drug targets, and predict therapeutic effects. This study investigates the generation of drug-gene interactions in periodontal bone resorption using VAEs.

Methods

A bone resorptive drugs dataset was retrieved from Probes and Drugs and analyzed using Cytoscape (https://cytoscape.org/) and CytoHubba (https://apps.cytoscape.org/apps/cytohubba), powerful tools for studying drug-gene interactions in bone resorption. The dataset was then prepared for matrix representation, with normalized input data. It was subsequently divided into training, validation, and testing sets. We then built an encoder-decoder network, defined a loss function, optimized parameters, and fine-tuned hyperparameters. Using VAEs, we generated new drug-gene interactions, assessed model performance, and visualized the latent space with reconstructed drug-gene interactions for further insights.

Results

The analysis revealed the top hub genes in drug-gene interactions, including Matrix Metalloproteinase (MMP) 14, MMP 9, HIF1A, STAT1, MAPT, CAS9, MMP2, CASP3, MMP1, and MAK1. The VAE's reconstruction accuracy was measured using mean squared error (MSE), with an average squared difference of 0.077. Additionally, the KL divergence value was 2.349, and the average reconstruction log-likelihood was -246.

Conclusion

The generative variational encoder model for drug-gene interactions in bone resorption demonstrates high accuracy and reliability in representing complex drug-gene relationships within this context.

## Introduction

Periodontal bone resorption is a major dental issue causing tooth loss and impaired oral function [1]. It is a multifactorial condition influenced by bacterial plaque, genetic predisposition, smoking, systemic diseases, medications, hormonal changes, and poor oral hygiene. The pathogenesis of periodontal bone resorption involves a complex interplay of inflammatory mediators, host immune response, and enzymatic activities. Bone remodelling plays a crucial role in maintaining the homeostasis of periodontal tissues, but in periodontal diseases, this balance is disrupted, favouring resorptive processes. Inflammatory mediators, such as prostaglandins, matrix metalloproteinases, and the receptor activator of nuclear factor kappa-B (RANK)*/*receptor activator of nuclear factor kappa-B ligand (RANKL)*/*osteoprotegerin​​​​​​​ (OPG) pathway, play a role in bone resorption [2] Novel management approaches, such as adjunctive therapies, antibiotics, host modulation agents, regenerative techniques, and minimally invasive surgical interventions, are expected to transform the management of periodontal bone resorption [3].

Osteoclasts, derived from hematopoietic stem cells, play a key role in bone resorption. In diseases like rheumatoid arthritis, periodontitis, and osteoporosis, there is an imbalance favouring bone resorption. Understanding the mechanisms of osteoclast formation and bone resorption is crucial. Osteocytes secrete RANKL, which is essential for osteoclast formation. Tumor necrosis factor-alpha​​​​​​​ (TNF-α) enhances RANKL expression in osteocytes and promotes osteoclast formation [4].

Additionally, TNF-α increases sclerostin expression in osteocytes, further promoting osteoclast formation. These findings indicate that osteocyte-related cytokines directly enhance bone resorption. This review discusses the role of osteocytes as the master regulators of bone resorption and effectors in osteoclast formation. Osteocytes play a crucial role in bone turnover and homeostasis by regulating osteoclast and osteoblast activity through RANKL signalling. They express RANKL significantly more than osteoblasts, influencing osteoclast differentiation and bone resorption. Additionally, osteocytes can affect osteoblasts through signals like sclerostin and DKK1, which impact Wnt/β-catenin signalling in osteoblast formation and matrix development [5].

Periodontitis tissue contains pathological factors such as biologically active substances in bacterial plaques and inflammatory mediators. These factors increase RANKL expression in osteocytes. Lipopolysaccharide​​​​​​​ (LPS) from gram-negative bacteria activates toll-like receptor (TLR) 2 on the osteocyte surface, leading to upregulation of interleukin (IL)-6 expression. TNF-α binds to the TNF receptor on the osteocyte surface, activating signalling pathways that increase RANKL expression and promote alveolar bone resorption. The RANKL/OPG ratio increases in periodontitis, leading to an enlarged bone resorption area. Osteocytes regulate RANKL trafficking to osteoclast precursors, and TNF-α enhances Sclerostin expression via a nuclear factor kappa-light-chain-enhancer of activated B cells​​​​​​​ (NF-κB) dependent mechanism. Osteocytes, especially those lacking RANKL, protect against bone loss in LPS-induced periodontitis​​​​​​​ (LIP), while RAG1-deficient mice do not. Osteocytes express NOD1 and induce more RANKL than osteoblasts, and LIP induces bone resorption and formation [6].

Drug-gene interactions occur at various biological complexity levels, including protein complexes, protein-protein families, and individual proteins. These interactions play a crucial role in modulating cellular processes and can be targeted by drugs for therapeutic effects. Drugs can disrupt specific protein complexes, target protein-protein families, or directly bind to specific proteins, affecting protein function, signalling pathways, and cellular processes. Understanding these interactions is essential for developing targeted therapies, identifying potential drug targets, and optimizing therapeutic approaches for various diseases and conditions [7].

Generative variational autoencoders (VAEs) can help model drug-gene interactions. They can generate new data by training on existing datasets, extracting relevant features, aiding in drug discovery, and predicting drug-gene interactions. VAEs can also help identify potential drug targets and therapeutic interventions by learning patterns and relationships in existing data. They can also be used for predictive modelling, evaluating the therapeutic effects of specific drugs on specific genes.

This work proposes using VAEs as a machine-learning approach to model and generate drug-gene interactions in periodontal bone resorption. VAEs, a deep generative model, can learn the underlying distribution of drug-gene interactions from available data. Training the VAE on a large dataset of known drug-gene interactions can teach shared patterns and relationships between variables. The trained VAE can generate new drug-gene pairs in periodontal bone resorption, potentially uncovering previously unexplored interactions. This approach offers several advantages, including a data-driven approach to understanding the complex relationships between drugs and genes, generating new drug-gene pairs, and personalizing treatment options based on individual patient characteristics. This study aims for generative drug-gene interactions in periodontal bone resorption using variational autoencoders

## Materials and methods

Dataset preparation

Using Probe & Drugs [8], the bone resorptive drugs dataset was downloaded and retrieved, and missing values and outliers were removed, and the dataset contains columns like 'pdid,' 'name,' 'gene_name,' 'target_type,' 'moa,' 'human,' 'activity_biochemical,' 'activity_cell,' 'probe.'

Cytoscape

Cytoscape (https://cytoscape.org/) and cytoHubba (https://apps.cytoscape.org/apps/cytohubba) [9] are powerful tools for studying drug-gene interactions in bone resorptive drugs. Cytoscape is an open-source software platform for visualizing and analyzing biological networks, while cytoHubba is a plugin designed for network analysis and identifying hub genes or proteins. These tools help construct biological networks based on known interactions between drugs and genes involved in bone resorption. CytoHubba uses various algorithms to identify hub genes, which can be used as potential therapeutic targets. Data were retrieved into Cytoscape, a drug-gene interactome was built, and hub genes were identified using cytoHubba with the Maximum Clique Centrality (MCC) method.

Methods for variational autoencoders

A dataset was prepared for a matrix representation, normalized input data, divided into training, validation, and testing sets, built an encoder and decoder network, defined a loss function, optimized parameters, fine-tuned hyperparameters, generated new drug-gene interactions, assessed model performance, and visualized the latent space with reconstructed drug-gene interactions for insights. These steps are necessary to train a VAE to use a Python environment for drug-gene interaction modelling.

Generative variational autoencoders architecture and hyperparameter

Variational autoencoders (VAEs) are deep learning models that learn the distribution of a dataset using an encoder network, decoder network, and loss function. Their architecture and hyperparameters significantly impact their performance and quality of samples.

The encoder network in a VAE uses input data, like drug-gene interaction modeling, through neural network layers to reduce data dimensionality, producing the mean and variance of a multivariate Gaussian distribution for point sampling. The decoder network maps input to input space, reversing the structure of the encoder network. It gradually increases data dimensionality until it matches the original input shape, with configurable hyperparameters like layers and neurons per layer.

The loss function in VAEs consists of a reconstruction loss and a regularization term. The reconstruction loss measures the decoder network's ability to reconstruct original input data, while the regularization term penalizes deviation from a standard Gaussian distribution, encouraging similar input data mapping. The hyperparameters of a VAE, including architecture and regularization terms, are crucial for performance. The model's learning capacity, including layers and neurons and activation functions sigmoid, also impacts its performance. The architecture, loss function, and regularized hyperparameters were fine-tuned to balance latent space accuracy and regularization. The KL divergence term can be weighted to control exploration, while learning rate influences step size, training speed, and stability. Hyperparameter selection requires experimentation and tuning, using grid search, random search, and cross-validation to explore hyperparameter space and optimize configurations. The architecture and hyperparameters of variational autoencoders significantly influence their learning capacity, reconstruction quality, and latent space exploration, making them useful in drug-gene interaction modelling and other generative tasks.

## Results

Drug-gene interactome

**Figure 1:**
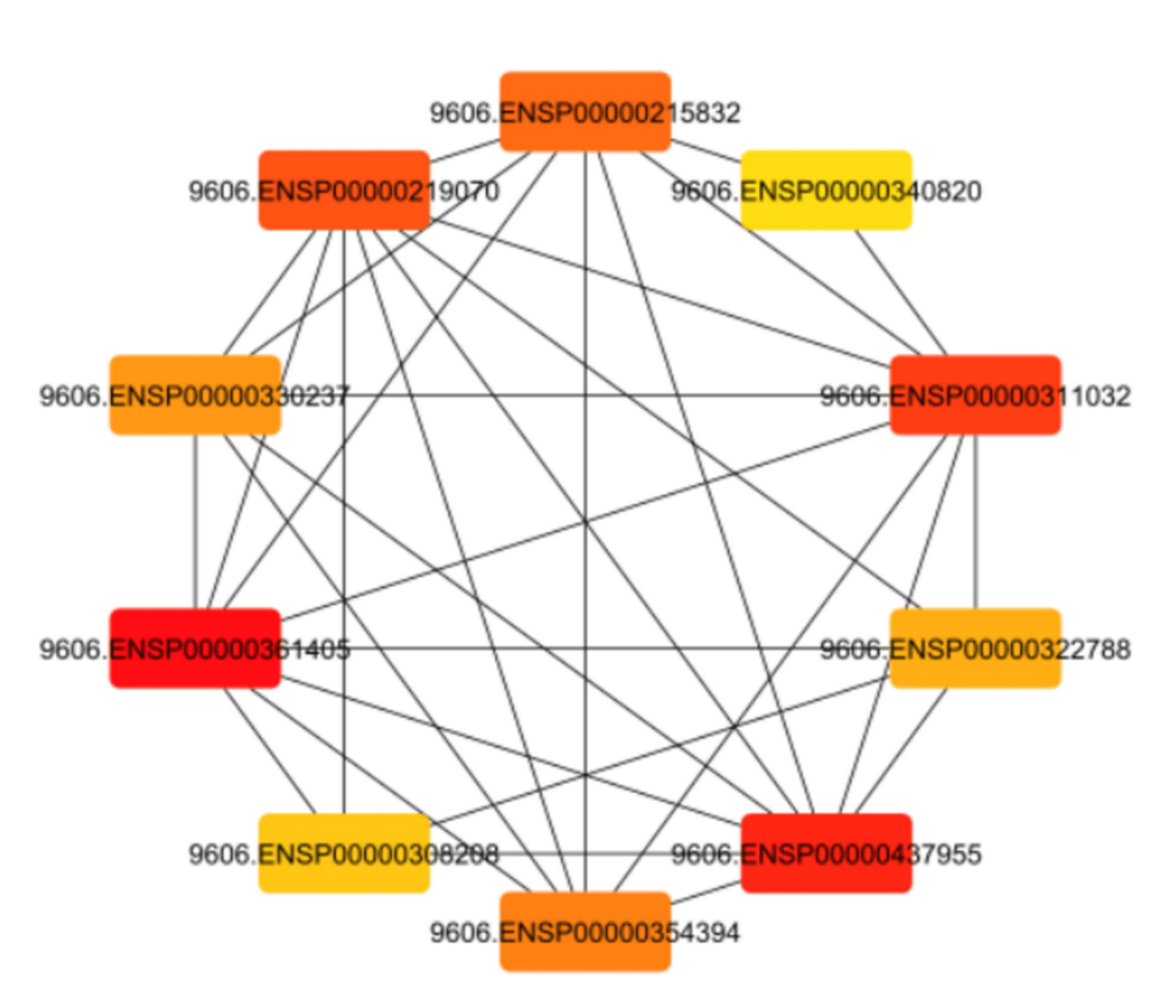
Top hub genes in drug-gene interactions

Network interactome provides information on a network with 83 nodes and 106 edges. The network has an average of 2.568 neighbours, a network diameter of 10, a radius 5, and a characteristic path length of 4.624. The clustering coefficient measures the degree of interlinking between nodes. The network density is 0.032, and the network heterogeneity is 1.279. The network centralization is 0.223, and there are two connected components. The analysis time is 0.059 seconds, and the network has 106 edges (Figure 1). Top hub genes in drug-gene interactions were MMP-14, MMP9, HIF1A, STAT1, MAPT, CAS9, MMP2, CASP3, MMP1, MAK1.

**Figure 2:**
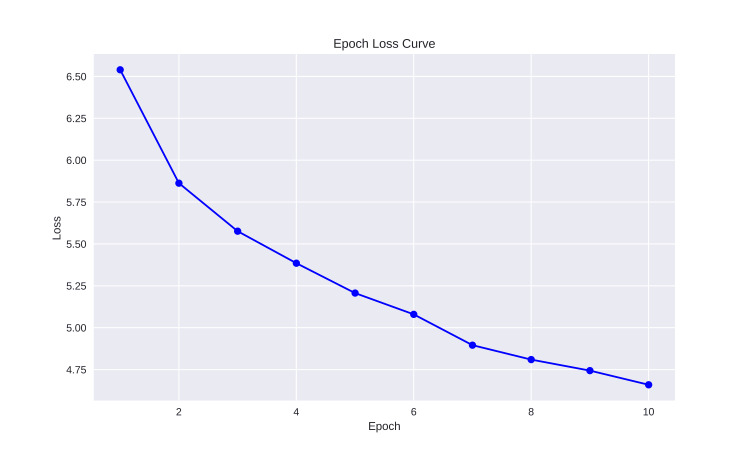
Epoch loss curve

Figure 2 shows an "Epoch Loss Curve," illustrating the correlation between loss values and epoch numbers in a machine learning model training process. The x-axis represents the epochs from 1 to 10, while the y-axis represents the loss values ranging from just above 6.5 to below 5. The graph shows a decreasing trend, indicating that the loss decreases as the number of epochs increases. The graph, with data points connected by lines, is commonly used to monitor and analyze machine learning model performance during training. The training logs for each epoch include losses in epochs 1, 2, 3, 4, 5, 5, 6, 7, 8, 8, 9, and 10, with average losses ranging from -0.5 to -4.6. The model's average loss for each epoch is 4.65.

Epoch loss curve

The loss curve in VAEs shows a steady decrease in loss over epochs, indicating the model's learning and improvement in reconstruction ability. The initial significant drop in loss is typical as the model quickly learns the data's structure. As training progresses, the rate of decrease slows down, suggesting the model is converging towards a local minimum in the loss landscape. This indicates the model is refining its representation and making smaller adjustments to improve reconstruction accuracy. The decreasing loss curve demonstrates the VAE model's effective learning and improvement in reconstructing input data as training progresses.

Reconstruction mean squared error (MSE)

Reconstruction Mean Squared Error (RMSE) is a metric used to measure the performance of a reconstruction model, such as regression or image reconstruction. It quantifies the average squared difference between predicted and actual values. The formula for RMSE is: RMSE = sqrt( (1 / N) * sum((predicted_i - actual_i)^2)). A lower RMSE value indicates better performance, as predicted values are closer to actual values. However, RMSE alone may not provide a complete evaluation, so it is often used with other metrics.

The mean squared error (MSE) of 0.07769 indicates that the Variational Autoencoder (VAE) model effectively learns the underlying patterns and structures in the data, resulting in better reconstruction quality. This indicates that the VAE model accurately generates reconstructions that closely resemble the original data points, demonstrating its ability to reconstruct input data from latent space accurately.

Reconstruction accuracy

Reconstruction accuracy in a VAE model refers to the model's ability to reconstruct or reproduce input data, measured by metrics like mean squared error (MSE). Higher accuracy indicates a model's ability to capture and reproduce original data points accurately. Other metrics like MAE, PSNR, and SSIM can also be used. Reconstruction accuracy depends on architectural design, training process, hyperparameters, and input data quality. Balancing accuracy with other performance aspects is crucial.

 The VAE's reconstruction accuracy is measured using the MSE, with an average squared difference of 0.077, indicating good performance. However, there is room for improvement as the MSE is not zero, indicating a potential loss of information. Improving the accuracy could involve adjusting the model architecture, hyperparameters, or training data. The quality and representativeness of the input data also influence the accuracy. Careful analysis and experimentation are crucial to achieve the desired level of accuracy.

Reconstruction log-likelihood

The reconstruction log-likelihood is a metric used to evaluate the quality of reconstructions generated by a variational autoencoder (VAE). It quantifies the probability of the original data given the reconstructed data. A higher log-likelihood indicates better reconstruction, while a lower one suggests difficulty. The VAE aims to minimize log-likelihood during training to improve its performance. Other metrics like KL divergence and perceptual similarity can also provide insights.

The VAE's average reconstruction log-likelihood of -246 indicates that it produces reconstructions that closely match the original input data. The reconstruction log-likelihood measures the VAE's ability to reconstruct original data, with higher values indicating better quality. The model achieved a lower negative log-likelihood, preferring a lower value. Interpretation depends on the dataset and problem context, and comparisons are useful.

KL divergence

KL Divergence, also known as Kullback-Leibler Divergence, measures the difference between two probability distributions. It is used in information theory and statistics to compare distributions. KL divergence is a non-negative quantity, with 0 indicating identical distributions and higher values indicating greater dissimilarity. In machine learning and generative models like Variational Autoencoders, it is used as a regularization term to encourage the learned latent space to have a similar distribution as the prior distribution, balancing reconstruction quality with sample diversity. The average KL divergence value is 2.349, representing the average difference between two probability distributions. Understanding its significance requires context and understanding the specific probability distributions being compared and the problem being analyzed.

## Discussion

Anti-resorptive drugs treat periodontal bone loss by inhibiting osteoclast activity. Studying drug-gene interactions can identify genetic factors influencing response to these medications and tailor treatment strategies. The hub genes you listed, MMP-14, MMP9, HIF1A, STAT1, MAPT, CAS9, MMP2, CASP3, MMP1, and MAK1, have been implicated in various aspects of periodontal bone resorption. 

Matrix metalloproteinases (MMPs), specifically MMP-14 and MMP9, are involved in the breakdown and remodeling of the extracellular matrix, particularly during bone resorption [10,11]. MMP-14, a cell membrane protein, activates proMMP-13 to break down type I collagen, a key component of radicular cement, periodontal ligament, and alveolar bone matrix. It also self-activates through self-proteolysis, influenced by reactive oxygen species [12,13]. Hypoxia-inducible factor-1 alpha (HIF-1α) is a transcription factor that regulates gene expression, bone resorption, and angiogenesis. It involves bone resorption and periodontitis, causing gingival inflammation and bone loss. Substance P upregulates HIF-1α levels, promoting osteoclast differentiation [4,14].

STAT1 [5,15] is a transcription factor involved in immune responses and osteoclast differentiation. It controls osteoclast activity and is involved in the exacerbated periodontitis caused by Nos3-/-related hypertension. STAT1 inhibitors can reduce proinflammatory cytokine expression and macrophage infiltration. Nos3-/- hypertension downregulates STAT3's anti-inflammatory function and downstream chemokine expression. Microtubule-associated protein tau (MAPT) is linked to neurodegenerative diseases and bone metabolism, with dysregulation of MAPT expression potentially causing abnormal bone remodeling. The study highlights the complex network of interactions between hub genes and other genes and signaling pathways, highlighting their potential as therapeutic targets and the need for further research.

Drug Property Identification (DPI) approaches include docking-based, machine learning-based, and deep learning-based methods. Docking involves finding protein structures, while machine learning requires manual features [7,16]. Deep learning improves prediction performance by analyzing network parameters and structure. The variational autoencoder (VAE) is a machine learning model used in image and text processing to predict drug-protein interactions [6,7,16]. It reduces redundant information, extracts discriminative local features using deep Convolutional Neural Network (CNN), and performs robust identification on various datasets, demonstrating its potential in drug-protein interactions. Latent representations of drugs and their targets produced by contemporary graph autoencoder-based models have proved useful in predicting many types of node-pair interactions on large networks, including drug-drug, drug-target, and target-target interactions. However, most existing approaches model the node's latent spaces in which node distributions are rigid and disjoint; these limitations hinder the methods from generating new links among pairs of nodes. A recent study discusses the effectiveness of variational graph autoencoders (VGAE) in modeling latent node representations on multimodal networks. It suggests a new method concatenating Morgan's fingerprints with latent embeddings for link prediction [15]. The model shows good accuracy and competitive results on drug and protein and cell line networks similar to this study's accuracy of The VAE's reconstruction accuracy is measured using the MSE, with an average squared difference of 0.077, average KL divergence value of 2.349 and The VAE's average reconstruction log-likelihood of -246. (Figures 1, 2)

Variational autoencoders (VAEs) can study drug-gene interactions related to bone resorption, capturing relationships between drugs and genes and discovering new associations. However, there are several future directions and limitations to consider. These include incorporating additional data sources like genomic and clinical data or interpreting latent variables regarding biological or chemical factors that could provide insights into drug-gene relationships. Handling imbalanced datasets effectively is crucial to avoid biased predictions [17,18]. Incorporating uncertainty estimation techniques like Bayesian Variational Autoencoders can provide probabilistic uncertainty estimates for more reliable decision-making. Scaling to larger datasets is also essential for real-world scenarios. Addressing data sparsity and missing values improves the model's performance. Validation and reproducibility are essential for establishing the model's generalizability and reliability.

## Conclusions

The drug-gene interactions generative VAE model for bone resorption shows high accuracy and reliability in reconstructing input data. Its low MSE value, KL divergence value, and high reconstruction log-likelihood indicate its effectiveness in accurately representing complex drug-gene relationships in periodontal bone resorption.
